# Opportunities for Shared Decision-Making in the Management of Acute Pain in Asia-Pacific Countries

**DOI:** 10.7759/cureus.95305

**Published:** 2025-10-24

**Authors:** Kok Yuen Ho, Dinesh Nagrale, Pornthip K Chuchai, Jose Rhoel De Leon, Lourdes J Koh-Cabaluna, Sabarul A Mokhtar, Termphong Phorkhar, Jose B Rafanan, Gopinathan Raju, Passakorn Sawaddiruk, Prakrit Suwanpramote, Marvin Thepsoparn, Edward H Wang, Ankur Gupta

**Affiliations:** 1 Pain Management and Anaesthesiology, The Pain Clinic, Mount Alvernia Medical Centre, Singapore, SGP; 2 Medical Affairs, A. Menarini Asia-Pacific Holdings Pte Ltd, Singapore, SGP; 3 Pain Management, Fort Prachaksinlapakom Hospital, Udon Thani, THA; 4 Surgery, Capitol University Medical Center, Cagayan de Oro, PHL; 5 Pain Management, St Luke’s Medical Center, Quezon City, PHL; 6 Orthopaedics and Traumatology, Faculty of Medicine, Universiti Kebangsaan Malaysia, Hospital Canselor Tuanku Muhriz, Kuala Lumpur, MYS; 7 Pain Management, Chulabhorn Hospital, Bangkok, THA; 8 Pain Management, The Medical City Ortigas, Manila, PHL; 9 Anaesthesiology, Pantai Hospital Kuala Lumpur, Kuala Lumpur, MYS; 10 Anaesthesiology, Chiang Mai University, Chiang Mai, THA; 11 Orthopaedics, Faculty of Medicine, Ramathibodi Hospital, Bangkok, THA; 12 Anaesthesiology, Pain Management Research Unit, Faculty of Medicine, King Chulalongkorn Memorial Hospital, Chulalongkorn University, Bangkok, THA; 13 Orthopaedics, University of the Philippines - Philippine General Hospital, University of the Philippines, Manila, PHL

**Keywords:** low back pain, pain management, patient-centered care, patient centricity, postoperative pain

## Abstract

This expert elicitation involving nine key opinion leaders from Malaysia, the Philippines, Singapore, and Thailand aimed to describe current gaps in post-operative pain (POP) and low back pain (LBP) management in Asia-Pacific (APAC) countries and provide recommendations for the adoption of shared decision-making in acute pain management. Gaps in acute pain management due to patient or healthcare professional (HCP) challenges were identified. Patient challenges include inadequate understanding of the consequences of poorly managed acute pain, the goals of pain management and how to manage acute pain at home, as well as non-adherence to pain medications. Additionally, HCPs may have limited time to discuss pain management and treatment goals with patients or insufficient training in how to assess and manage acute pain. Several solutions based on shared decision-making principles were identified. These include training HCPs in pain management to support timely assessment, diagnosis, and treatment, as well as best practices for effectively communicating information to patients (e.g., using the Seek, Help, Assess, Reach, Evaluate (SHARE) approach). Other solutions include implementing tools for assessing multiple aspects of patient pain, improving public awareness of available treatments, and the provision of accessible learning materials to improve patient understanding of POP, LBP, and available management options. Furthermore, adopting a multidisciplinary approach that fosters HCP collaboration and addresses the full pain experience may lead to more accurate diagnoses and improved treatment outcomes. Shared decision-making offers a solution to the current gaps in acute pain management in APAC countries. Educating patients and training HCPs will be key to achieving a mutual understanding of symptoms and available treatment, the basis of shared decision-making, potentially improving clinical outcomes.

## Editorial

Postoperative pain (POP) and low back pain (LBP) are common, debilitating conditions affecting most adults worldwide, with LBP particularly prevalent during the later stages of life [1-5].

Postoperative pain is an often overlooked potential outcome of surgery among physicians, despite being a common preoperative concern for patients [6,7]. Over the past decade, substantial efforts have been made to increase access to surgery in the Asia-Pacific (APAC) region, in large part driven by the 2015 World Health Assembly resolution to strengthen emergency and essential surgical care [8,9]. As a result, the prevalence of POP in the APAC region is expected to increase. In a 2023 Chinese study, almost 60% of patients experienced moderate to severe POP in the first 24 hours after surgery, and this pain continued for more than 24 hours post-surgery in more than a third of cases [10].

LBP is a leading cause of disability and is among the most common musculoskeletal conditions [2,11]. Prevalence of LBP is increasing, with South Asia expected to experience some of the largest increases in cases globally [2]. In 2020, approximately 117 million individuals were affected by LBP in South Asia, and this figure is expected to increase by more than half to 176 million by 2050, driven by a growing aged population [2].

Both POP and LBP are associated with a substantial quality-of-life burden. Patients experiencing POP or LBP can be affected by a range of adverse consequences including increased morbidity, impaired physical function and quality of life, increased recovery times, prolonged opioid use during and after hospitalization, and greater healthcare costs [3,12].

Historically, healthcare in the APAC region has been largely based on a paternalistic approach, characterized by physicians assuming the dominant role in the physician-patient relationship [13-16]. Factors contributing to this precedent include educational gaps between patients and physicians, a lack of partnership communication training for physicians and limited time for implementation, high patient load per physician, limited patient engagement due to cultural or religious beliefs, and economic factors relating to the widely adopted fee-for-service healthcare model [13-15].

Optimal pain diagnosis and treatment can be challenging and rely on physicians receiving an accurate description of pain levels from patients, which are subjective and often differ between patients [17]. In the APAC region, there exist additional challenges to optimal pain management beyond those encountered in other parts of the world. An important consideration relates to prevalent patient cultural beliefs that can influence the pain management decision-making process. In many Asian cultures, patients may not engage with physicians for management of LBP or POP due to a perception of pain being a normal part of life [18]. Additionally, chronic pain is not managed optimally in low- and middle-income countries where there exist limited resources or capacity to deliver non-pharmacological care for LBP [18]. Furthermore, limited access to analgesics and a lack of awareness and knowledge about pain management strategies among patients and physicians are also challenges that must be addressed to improve outcomes in APAC countries [18].

In Western countries, shared decision-making between patients and physicians is widely regarded as the ideal model for making disease management choices, as it is associated with improvements in the quality of decisions, patient outcomes, risk perception, patient adherence, and autonomy [19-22]. In this model, physicians use their training, knowledge, and experience to provide patients with information and context to support their diagnosis, with patients’ needs, preferences, and values taken into account [23,24]. The shift towards shared decision-making is largely driven by modernization and the growing digital health literacy among younger patients, which has heightened their demand for involvement in healthcare decisions and enhanced their capacity to engage with health information [25]. The implementation of a shared decision-making approach has led to improved clinical outcomes (e.g., patient knowledge, satisfaction, treatment adherence, and engagement) in several therapeutic areas [26-31].

Despite the benefits of shared decision-making, this approach is not widely adopted in clinical practice. Specifically, in acute pain management, the incorporation of shared decision-making practices has been slow to emerge, and current guidelines do not provide recommendations on implementing shared decision-making into clinical practice [24,32,33]. However, studies show that shared decision-making has the potential to optimize acute pain management by providing higher quality care and improving patient outcomes [24,34]. A real-world study comparing POP management outcomes among patients treated in Chinese (n=268) and US (n=244) hospitals found that Chinese patients received less information about POP and were less involved in pain management decisions compared with US patients [35]. Consequently, Chinese patients achieved significantly inferior outcomes including pain severity, interference with activity, affective experiences, adverse effects, and perception of pain care (all p<0.001) compared with US patients [35].

Given the opportunities for shared decision-making to improve patient outcomes in acute pain management, we set out the current gaps in acute pain management in APAC countries and recommendations for its adoption.

Patient journey

When identifying areas to incorporate shared decision-making in the management of acute pain, it is important to consider the patient journey. The term patient journey encompasses the clinical pathways for diagnosis and treatment and the emotional and behavioral experiences of the patient, including those prior to initial evaluation and diagnosis and after treatment [36]. This spans the entire patient experience, from symptom onset through continuous management to monitoring, follow-up, resolution, and recovery [36].

**Figure 1:**
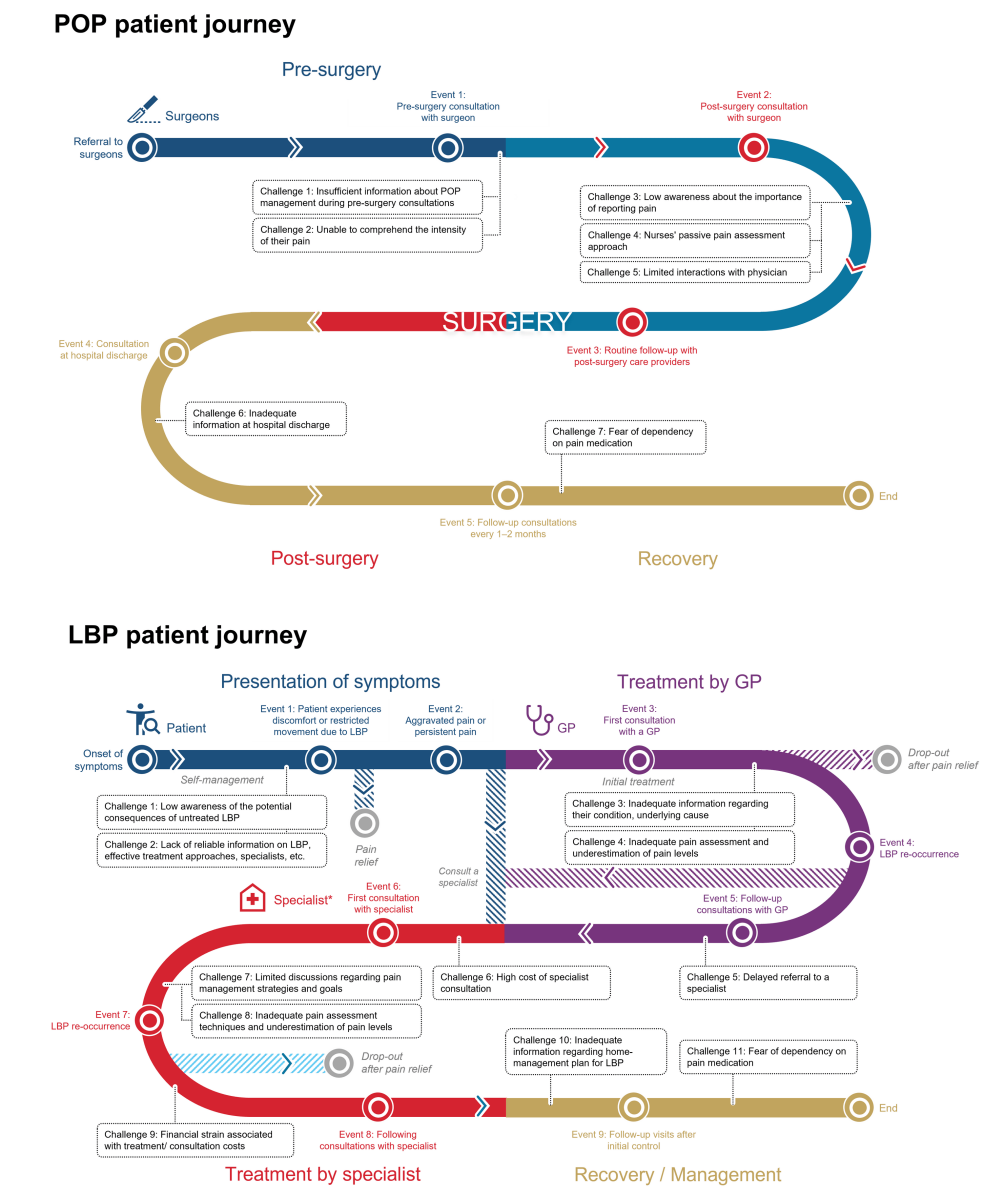
POP and LBP patient journey ^*^Orthopedics, obstetrician-gynecologist, etc. POP: Post-operative pain; LBP: low back pain

In POP, the patient journey can be categorized into three stages: pre­surgery, surgery and post-surgery, and recovery (Figure 1). Sample guidance for treatment recommendation at each stage of POP, proposed by Santos et al. [37], is provided in Table 1. In LBP, the patient journey can be divided into four stages: presentation of symptoms, treatment by a general practitioner (GP), treatment by a specialist, and recovery and management (Figure 1). These patient journeys were developed in discussion with nine key opinion leaders (KOLs) comprising five experts in POP and four in LBP. Participants included pain specialists, anesthesiologists, orthopedic surgeons, and general surgeons from Malaysia, the Philippines, Singapore, and Thailand.

**Table 1:** Sample guidance for treatment recommendation in the management of POP ^a^Adapted from APS, ANZCA and the PROSPECT initiative with permission from Santos et al. [37]. ^b^The list does not contain all molecules available within a therapeutic class. ^c^IV Hydromorphone is not available in the Philippines; similarly, not all therapeutic options will be available in all countries. ANZCA, Australian and New Zealand College of Anaesthetists; APS, American Pain Society; FDC, fixed-dose combination; IV, intravenous; NPO, nothing by mouth; NSAID, non-steroidal anti-inflammatory drugs; PROSPECT, PROcedure-SPECific pain management; POP, postoperative pain. Source: Adapted from Santos et al. [37]

Time		Immediately postoperative		Postoperative		Discharge	
Severity		Moderate-to-severe pain				Mild-to-moderate pain	
Analgesia	Time	0–24 hours		Day 1–5		Day 6–10	
	Route	NPO (IV)		Oral		Oral	
	Guidelines^a^	Strong/weak opioid +/- NSAID		Strong/weak opioid +/- NSAID		NSAID +/- paracetamol	
Therapeutic options^b^		Strong/weak opioid (IV) Hydromorphone,^c^ Oxycodone, Morphine, Tramadol	NSAID (IV) Dexketoprofen, Ketorolac, Diclofenac, Parecoxib	Strong/weak opioid Tramadol (mono), Oxycodone Morphine, Tramadol/paracetamol (FDC)	NSAID Dexketoprofen, Diclofenac, Ibuprofen, Etoricoxib, Celecoxib	NSAID Dexketoprofen, Diclofenac, Ibuprofen, Etoricoxib, Celecoxib	Paracetamol
				Tramadol/dexketoprofen (FDC)			

Gaps in the management of POP

The management of POP remains challenging in APAC countries, with many patients experiencing insufficient pain control following surgery. Patient challenges identified by healthcare professionals (HCPs) are described and summarized in Table 2.

**Table 2:** Key gaps in the management of POP and LBP in APAC countries APAC, Asia-Pacific; GP, general practitioner; HCP, healthcare professional; LBP, low back pain; POP, postoperative pain.

Key gaps in the management of POP
Patient misconceptions around the consequences of insufficient POP management.
Inadequate information provided to patients at pre-surgery consultations on POP management goals.
Limited access to multidisciplinary care.
Low awareness of the importance of reporting pain among patients, and even some HCPs.
Insufficient time for physicians to discuss POP management plans and goals with patients.
Inadequate guidance provided to patients on home-management plans at hospital discharge.
Non-adherence to POP relief due to concerns about adverse effects and addiction.
Key gaps in the management of LBP
Lack of understanding or awareness of the repercussions of untreated LBP among the public, and even some HCPs.
Insufficient knowledge and guidance available to patients on symptoms, disease course, effective treatment approaches, and appropriate specialists.
Delays in specialist referrals due to inadequate pain assessments or mis-referrals by GPs.
Limited time for specialists to discuss LBP management, treatment strategies, and goals with patients.
Insufficient information provided to patients on long-term home-management plans.
Non-compliance to pain management regimens due to fears about addiction and undesirable side effects.

At the early stages of the patient journey, there is a general lack of awareness among patients of the potential consequences of inadequate pain management after surgery. This is due to insufficient information provided to patients during pre-surgery consultations, forcing many patients to rely on online resources (e.g., Facebook, YouTube) as their main sources of information (Meeting report: Yuen HK, Chuchai PK, Leon JRD, Koh-Cabaluna LJ, Mokhtar SA, Nagrale D, Phorkhar T, Rafanan JB, Raju G, Sawaddiruk P, Suwanpramote P, Thepsopar M, Wang E. AURORA Expert Meeting on Patient Centricity in Pain Management; March 6, 2024) [38]. Key information gaps include the goals of POP management and the potential risks and complications of poor POP management. Consequently, limited knowledge relating to POP can cause some patients to delay surgery due to the fear of pain or being unable to return to work after the surgery.

Multidisciplinary care offers benefits such as early and accurate diagnosis, rapid initiation of treatment following diagnosis, and potential improvements in patients’ quality of life (Meeting report: Yuen HK, March 6, 2024) [39]. However, a treatment gap exists in APAC countries such that multidisciplinary care (e.g., pain specialists, physiotherapists) for POP is typically only available in a relatively small number of leading hospitals, despite being the recommended approach (Meeting report: Yuen HK, March 6, 2024). Potential reasons for this include a lack of knowledge sharing among hospitals and the absence of specialized pain units in rural hospitals.

At the post-surgery stage, low awareness of the importance of reporting pain among patients and HCPs can be a barrier to optimal POP management (Meeting report: Yuen HK, March 6, 2024). Patients undergoing their first surgery tend to downplay pain; this is linked to inadequate information about the necessity of reporting POP for better functional recovery, shorter hospital stays, and lower incidence of chronic pain (Meeting report: Yuen HK, March 6, 2024). Nurses frequently adopt a passive approach in asking patients about their pain levels, and many do not offer sufficient advice on how to manage it (Meeting report: Yuen HK, March 6, 2024). Failure to adequately assess moderate pain levels at the hospital often results in patients being discharged with routine pain medicines that are insufficient to adequately address pain. As a result, patients either suffer at home or return to the hospital or their GP requesting additional pain relief (Meeting report: Yuen HK, March 6, 2024).

Furthermore, physicians often do not engage in discussions with patients on POP management plans or goals, and there is typically a lack of adequate information given at hospital discharge (Meeting report: Yuen HK, March 6, 2024). After surgery, physicians tend to prioritize disease recovery over pain management as they often perceive pain as a transient discomfort (Meeting report: Yuen HK, March 6, 2024). Consequently, when physicians deprioritize pain management, patients may underestimate the severity of their pain as they consider it inevitable and are therefore more likely to endure it (Meeting report: Yuen HK, March 6, 2024). Patients also have concerns about addiction and the adverse effects of pain medications, which can lead to poor treatment compliance (Meeting report: Yuen HK, March 6, 2024). In addition, patients are not always educated on at-home management at discharge, and if information is provided, it is usually verbally communicated, posing challenges for patients to remember and understand (Meeting report: Yuen HK, March 6, 2024).

Gaps in the management of LBP

Similar to POP, the management of LBP remains challenging in APAC countries, with many patients lacking clarity on the pain management pathway and experiencing inadequate pain control. Patient challenges identified by HCPs are described and summarized in Table 2.

In the early stages of the patient journey, a low general awareness of the consequences of untreated LBP among the public and even some HCPs can be a barrier to optimal pain management [18,40]. Patients with LBP often delay seeking medical advice, hoping the pain will go away, and only act once daily activities are severely impacted. Patients, and even some HCPs, often view LBP as a transient discomfort and may not grasp the full extent of its consequences or the necessity for prompt and accurate treatment [18]. Consequently, patients with musculoskeletal LBP typically endure pain for 1-4 years before seeking medical attention (Meeting report: Yuen HK, March 6, 2024). Most patients initially self-medicate, going through pharmacy channels or physical therapy before seeking medical attention (Meeting report: Yuen HK, March 6, 2024) [40]. The choice of self-medication varies by country; patients in the Philippines generally avoid over-the-counter (OTC) medicines and opt for topical gels, while in Thailand, OTC oral therapies are preferred (Meeting report: Yuen HK, March 6, 2024). The trigger to seek medical care is aggravated pain that is not addressed by OTC medications or other self-treatment methods.

Once the patient has sought medical advice, a lack of reliable information on symptoms, disease course, effective treatment approaches, and appropriate specialists imposes substantial barriers to accessing suitable treatment. Patients often conduct their own online research, which can lead to misinformation, delayed care, and worsening pain. Furthermore, currently available online information is often technical, limiting patient understanding of the condition and treatment options.

Delays in specialist referrals also present considerable challenges for patients. The first point of physician contact when patients seek medical attention for their pain is the GP. However, GPs often view LBP as a common complaint and may not conduct adequate pain assessments, often due to limited awareness or training in pain management [18]. Patients are only referred to specialists if pain persists for several months (average three months), or if pain escalates to severe levels despite treatment by the GP (Meeting report: Yuen HK, March 6, 2024). There is also low awareness among GPs of the appropriate specialist to refer for LBP, leading to potential mis-referrals and subsequent treatment delays. Patients who receive the correct referral usually receive treatment within 3-‍6 months, while patients who are mis-referred can experience treatment delays for up to 12 months (Meeting report: Yuen HK, March 6, 2024).

Once patients receive the appropriate specialist consultations, specialists often have limited time (typically a maximum of 10 minutes) to discuss pain management, treatment strategies, and goals with patients. Additionally, due to constraints in time and resources, specialists in a busy practice may not conduct a thorough assessment of pain levels and often underestimate the level of pain felt, leading to inadequate pain management treatment plans.

During the recovery and management stage of the patient journey, patients do not receive adequate information on long-term management plans, recovery trajectory, home-based management, and the importance of continuing follow-up visits with physicians to monitor pain levels and adjust treatment (Meeting report: Yuen HK, March 6, 2024). Consequently, some patients stop follow-up entirely and rely on OTC medicines due to the acceptable pain relief offered by these products and the financial strain associated with treatment or consultation costs (Meeting report: Yuen HK, March 6, 2024). Similar to POP, patients often refrain from continuing pain medication due to concerns with addiction and adverse effects (e.g., nausea, vomiting, and impaired renal function) (Meeting report: Yuen HK, March 6, 2024).

Shared decision-making in the management of POP and LBP

Shared decision-making empowers patients to make informed decisions, together with their physician, based on both clinical evidence and their individual preferences, beliefs, and values. Patient involvement in decision-making is associated with increased knowledge about their condition and treatment, higher patient satisfaction and treatment adherence, and can result in improved health outcomes [41-43]. Physicians who actively engage with patients in their care decisions benefit from an improved decision-making experience, an increase in the efficiency of care due to a reduced number of diagnostic tests and referrals, a higher patient retention, and a potential reduction in the risk of litigation [41-43].

**Figure 2:**
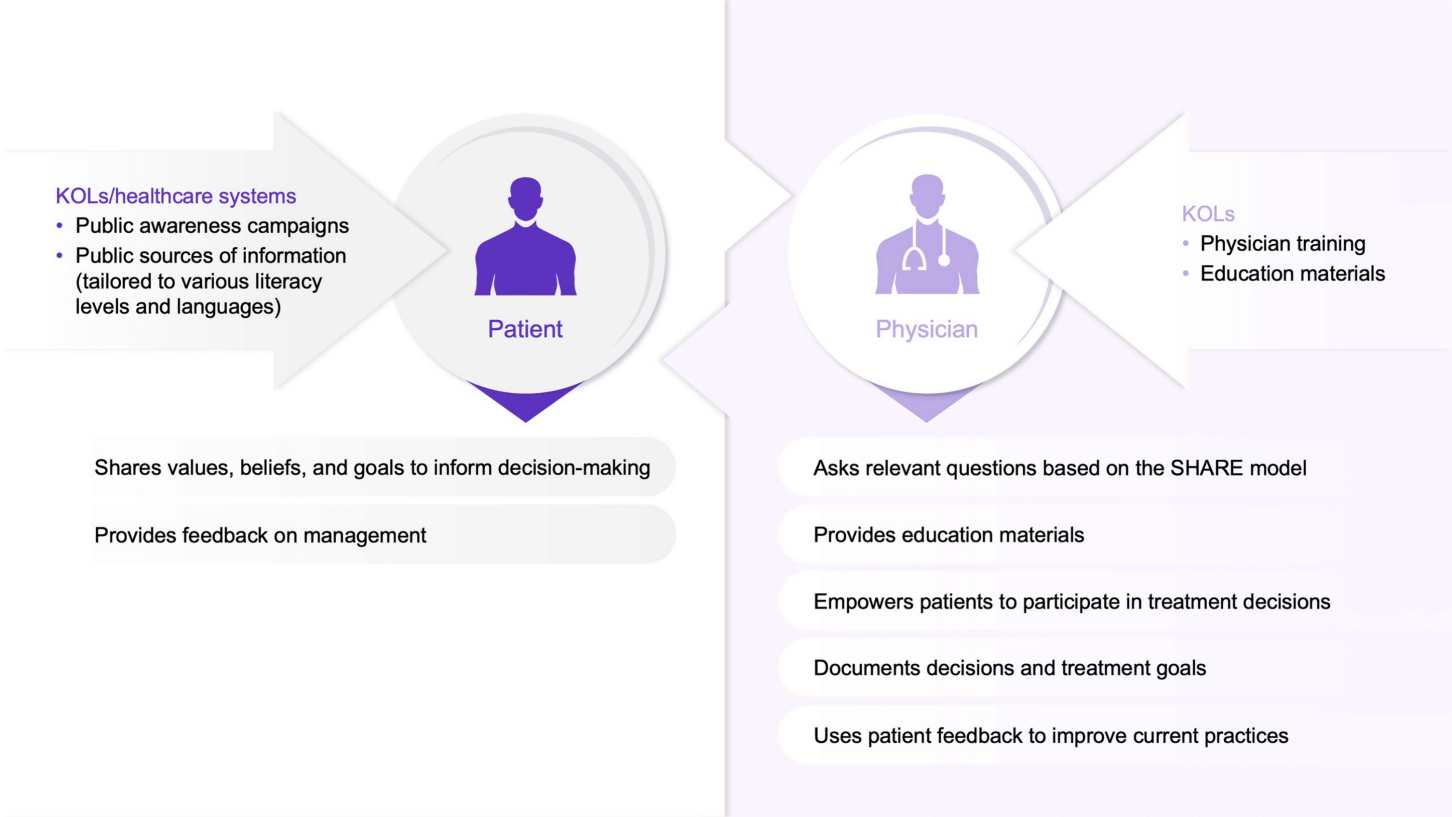
Opportunities for shared decision-making in the management of POP and LBP KOL, key opinion leader; LBP, low back pain; POP, postoperative pain; SHARE, Seek, Help, Assess, Reach, Evaluate.

We have highlighted opportunities for shared decision-making in acute pain management based on interviews with KOLs that aim to address current treatment gaps (Figure 2).

Patient engagement remains a longstanding challenge in pain management. Many patients would like their HCP to listen more closely to their pain experiences [44]. The initial medical visit is a pivotal moment in the POP and LBP treatment journey to establish the patient-physician relationship and build a mutual understanding of pain symptoms [24]. When decisions are made, patients have emphasized the importance of adopting a holistic view when diagnosing and treating pain, taking into account their needs, preferences, and values [44].

Low health literacy among patients and limited knowledge of their condition and available treatment options are key barriers to informed, shared decision-making. Patients may also have limited experience participating in healthcare decisions, and many do not recognize the importance of communicating their values when physicians make decisions around the management of their pain [45]. Patients may require additional education to better understand their pain and the treatment options available to them. Providing patients with background information in the form of leaflets and disease awareness websites catered to different literacy levels and languages may improve public health literacy and encourage active participation in healthcare decisions [46].

Failing to meet patients' expectations is a common challenge in acute pain management [24,47,48]. Defining patient expectations early in the treatment process is therefore important for achieving patient satisfaction [24,47,48]. Expectations of treatment for pain vary considerably by patient; it is therefore necessary for HCPs to ask the right questions and discuss realistic outcomes for treatment to avoid patient dissatisfaction [47]. Despite this, many physicians have limited time to discuss pain management, treatment strategies, and goals with patients (Meeting report: Yuen HK, March 6, 2024). Using a discussion model such as the SHARE (Seek, Help, Assess, Reach, Evaluate) approach can help HCPs quickly identify patients’ preferences, concerns, and treatment goals, helping to streamline identification of the most appropriate treatment option for the patient within the time limitations of a typical consultation (Meeting report: Yuen HK, March 6, 2024) [21,24].

Many patients report that inadequate information is provided to them regarding their pain management plan. However, before patient-centered care can be delivered to the patient, clear treatment goals should be established and communicated. Documenting the shared decision in writing will ensure both the physician and patient have clarity in the treatment decision and highlight any potential misunderstandings before treatment initiation. A physical copy of the treatment plan should be given to the patient to ensure that they (and their caregivers/families) are aware of their management plan and what to expect from treatment.

Ultimately, the shared decision-making process does not end at treatment completion after successful pain management. Reflecting on the perceived patient experience is necessary for improving the shared decision-making process. Physicians can benefit from patient feedback on the decision-making process and treatment experience, helping them adapt practices to improve pain management [24].

Solutions to support shared decision-making

**Figure 3:**
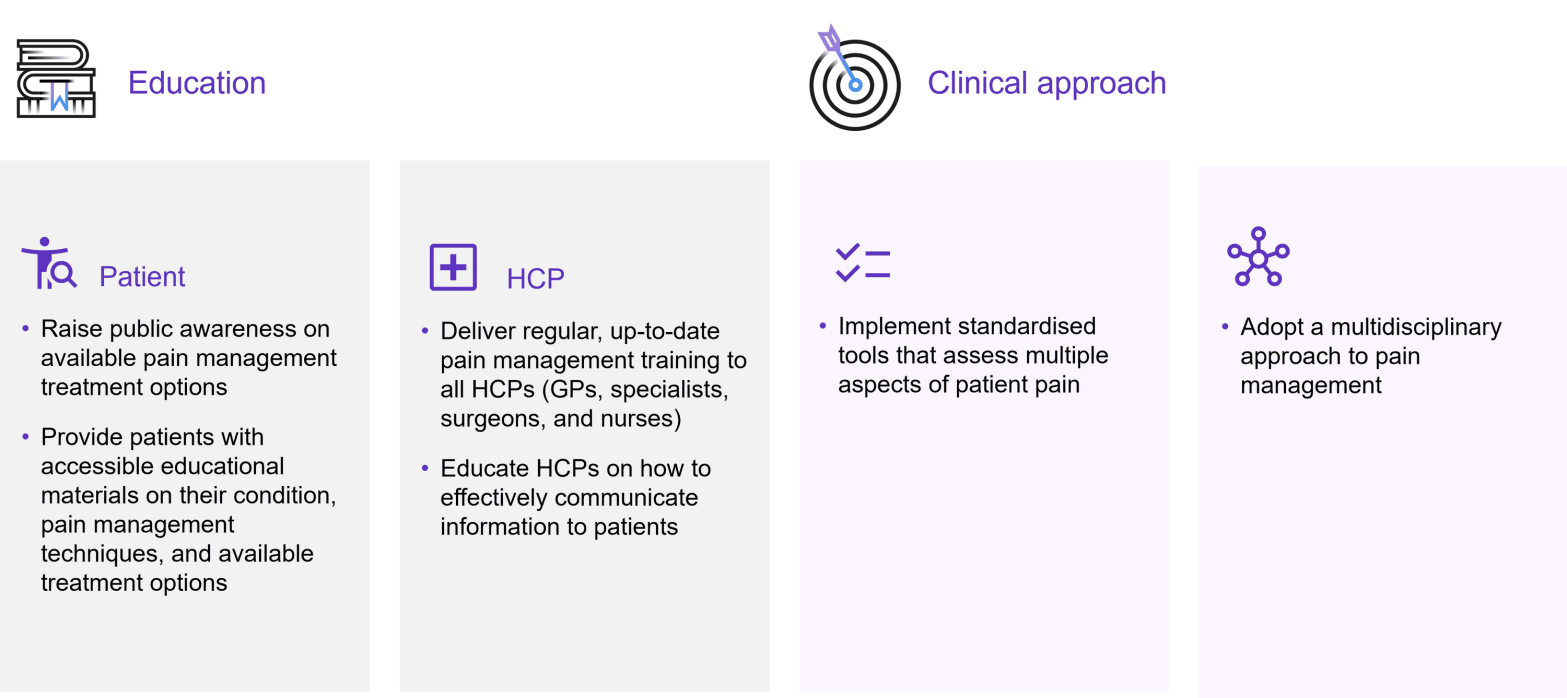
Key solutions to support shared decision-making GP, general practitioner; HCP, healthcare professional.

There exists a need for shared decision-making in acute pain management, driven by the diversity of underlying causes of pain, difficulties associated with patients’ individual understanding and experience of reporting pain, numerous treatment options available, and the need for timely treatment. Based on current evidence and expert opinion, we propose the following solutions to support greater use of shared decision-making in the management of POP and LBP (Figure 3 and Table 3).

**Table 3:** Key solutions that will support the implementation of shared decision-making to POP and LBP management in APAC countries APAC, Asia-Pacific; GP, general practitioner; HCP, healthcare professional; LBP, low back pain; POP, postoperative pain.

Key solutions that will support the implementation of shared decision-making in APAC countries
Including pain management as an integral part of training for all HCPs (GPs, specialists, surgeons, and nurses) will support timely specialist referral, diagnosis, and treatment.
Improving public awareness of available treatment options for pain management will cultivate a sense of urgency among the public to seek medical attention for their pain.
Providing patients with accessible educational resources (e.g., online materials or printed leaflets) that use local language and cultural context will enhance their understanding of the condition and available treatment options, which helps patients to actively participate in their treatment decisions.
Educating HCPs on what patients expect to know and how to share that information will support patients to be actively involved in shared decision-making.
Providing HCPs with regular KOL-led pain management training will facilitate a systematic approach for pain management with the most up-to-date practices.
Empowering nurses to communicate pain management plans to patients will ensure that patients receive the most up-to-date information, even in the absence of a physician visit.
Standardizing pain assessments with tools that consider multiple dimensions of the patient’s pain may allow for more timely and appropriate treatment.
Applying a multidisciplinary approach to pain management has the potential to deliver more accurate treatment diagnoses and improved treatment outcomes.

Education of HCPs and patients is key to increasing the use of shared decision-making in acute pain management. Pain is commonly under-recognized by physicians and often considered a transient discomfort. Pain management should therefore be included as an integral part of training for all HCPs (GPs, specialists, surgeons, and nurses). To make informed decisions about their condition and treatment, patients need accessible, easy-to-understand educational materials throughout their patient journey. In LBP, early diagnosis has been associated with improved outcomes, including improved quality of life and decreased risk of chronicity [49,50]. To support timely diagnosis and treatment, we advocate for cultivating a greater sense of urgency among the public for addressing LBP symptoms early. HCPs should engage with patient advocacy groups to emphasize the message that pain is curable and educate the public on available treatment options. Furthermore, raising awareness of effective pain management treatments may debunk patients’ preconceptions of opioids and correct opiophobia.

Once patients have sought medical attention for their pain, HCPs have a responsibility to facilitate informed decision-making by improving patients’ understanding of available treatment options and any side effects. To achieve this, we recommend that HCPs receive additional training on questions patients are likely to ask and how relevant information can be provided. Key topics of conversation should include treatment goals, plans, necessity of pain medications, and treatment/medicine-related information (e.g., side effects). Empowering patients to make informed decisions about their treatment enables physicians to choose the most suitable medication for the patient which they are most likely to adhere to. In the context of POP, we recommend that HCPs continue to educate patients post-surgery on pain management, as patients often rely heavily on information provided by their physician in the hospital.

Limited health literacy, low socioeconomic status, and language barriers have been recognized as substantial challenges for effective shared decision-making [51,52]. To ensure that information on pain management is accessible for all, we recommend providing education to patients in the form of written materials (e.g., leaflets) that use local language and cultural context. This also allows knowledge to be shared with caregivers and families, enabling them to be involved in the decision-making process.

Effective pain management is multifactorial and can be improved by adopting a systematic approach. To achieve this, KOLs should provide regular training to HCPs on the most up-to-date methods for pain control. In the context of LBP, training should highlight the nuances of LBP symptoms and signpost relevant key specialists to streamline referral. For POP, to ensure patients receive the most up-to-date information, even in the absence of a physician visit, we recommend that nurses are regularly educated in best practices for pain management and empowered to communicate this information to patients. Furthermore, we advocate standardizing pain assessment methods and moving away from a unidimensional numerical rating scale which does not capture the complexity and unique nature of the pain experience [53]. More timely and appropriate treatment may be achieved if multiple dimensions of the patient’s pain are considered.

Ultimately, collaborative action from KOLs, GPs, specialists, and healthcare systems is needed to support broader implementation of shared decision-making in acute pain management. Applying a multidisciplinary approach to pain management that encourages collaboration and communication among HCPs and ensures that all aspects of the patient’s pain experience are taken into consideration has the potential to deliver more accurate diagnoses and improved treatment outcomes.

Conclusions

Widely recognized gaps exist in the management of POP and LBP in the APAC region, which provides an opportunity for shared decision-making. Part of it involves educating patients on their condition, effective pain management techniques, and available treatment options. Training for HCPs (GPs, specialists, surgeons, and nurses) will also be key in shared decision-making to support a more systematic approach for optimal pain control and recovery. Driving widespread adoption of shared decision-making will require a multi-stakeholder approach, involving governments, professional bodies, and public health campaigns. Ultimately, the successful implementation of shared decision-making practices, built on informed conversations, mutual understanding of symptoms and treatment options, and greater collaboration between HCPs and patients, has the potential to improve clinical outcomes in acute pain management.
